# Does coping style mediate the relationship between knowledge and psychosocial outcomes in women with atrial fibrillation?

**DOI:** 10.3389/fpsyt.2024.1328111

**Published:** 2024-03-22

**Authors:** Michael R. Le Grande, Marielle Salvacion, Lubab Shwaita, Barbara M. Murphy, Alun C. Jackson, Marlies E. Alvarenga

**Affiliations:** ^1^ Australian Centre for Heart Health, Melbourne, VIC, Australia; ^2^ Melbourne Centre for Behaviour Change, School of Psychological Sciences, University of Melbourne, Melbourne, VIC, Australia; ^3^ Institute of Health and Wellbeing, Federation University, Berwick, VIC, Australia; ^4^ Faculty of Science, Engineering and Built Environment, Deakin University, Burwood, VIC, Australia; ^5^ Melbourne School of Psychological Sciences, University of Melbourne, Parkville, VIC, Australia; ^6^ Centre on Behavioral Health, University of Hong Kong, Hong Kong, Hong Kong SAR, China; ^7^ Victorian Heart Institute, Clayton, VIC, Australia; ^8^ Department of Psychiatry, School of Clinical Sciences, Monash University, Clayton, VIC, Australia

**Keywords:** atrial fibrillation, knowledge, coping, anxiety, depression, life satisfaction, mediation

## Abstract

**Introduction:**

In patients affected by atrial fibrillation (AF) disease-specific knowledge and coping style may be associated with psychosocial well-being. This study aimed to determine if coping style (problem-focused, emotion-focused, avoidance-focused) mediated the relationship between patient knowledge and three psychosocial outcomes (anxiety, depression and life satisfaction).

**Methods:**

In 2021 a total of 188 women with reported AF, and ages ranging from 18 to 83 years (mean 48.7, sd 15.5 years), completed an online questionnaire consisting of sociodemographic, clinical and AF knowledge questions and psychosocial instruments (Anxiety and depression, the Hospital Anxiety and Depression (HADS) scale; life satisfaction, Satisfaction With Life Scale (SWLS); and coping style (Brief COPE). Using Jamovi statistical software three individual mediational models (for anxiety, depression and life satisfaction) were constructed assessing the direct and indirect relationships between knowledge, coping style and each psychosocial outcome. Age was a covariate in each model.

**Results:**

The mediation analyses demonstrated significant direct negative associations between AF knowledge and HADS anxiety and depression and positive associations with SWLS. There were also direct associations between each of the three coping styles and the three psychosocial outcomes. There were significant indirect effects of coping style between AF knowledge and each of the three outcomes confirming partial mediation effects.

**Discussion:**

These findings highlight the crucial role of coping style in mediating the association between AF knowledge and psychosocial outcomes. As such, interventions aimed at increasing patient knowledge of AF may be more effective if adaptive problem-solving coping strategies are also demonstrated to these patients. Additionally, modification of maladaptive coping strategies as part of the psychological management of patients with AF is highly recommended.

## Introduction

Atrial fibrillation (AF) is the most common type of cardiac arrhythmia, affecting one in every four individuals at some point in their life (1). Cardiac arrhythmia refers to irregular heart rhythm and is caused by structural and electrical remodelling of the left atrium (2). The severity of AF ranges from brief paroxysmal episodes of electrical disturbance that terminate spontaneously within 48 hours to more regular episodes that last more than a week, and at the upper end of severity, into a permeant ongoing AF that causes cardiac remodelling, structural and electrophysiological remodelling of the tissue that provides substrates for maintenance of such arrhythmias, and functional impairment (3, 4). AF is a serious public health concern with an estimated prevalence of 5.35% in Australian adults over 55 years old (5). This is estimated to increase by 1.04% in the overall population aged >55 years by 2034 (5). In Scotland, Germany, and the USA, subjects aged 65 years or older show an AF incidence of 4.7, 4.1, and 28.3 per 1,000 person/years, respectively (6). Aside from age, 50% of AF cases can be explained by underlying risk factors that result in structural remodelling of the myocardium, including medical, demographic, psychological, behavioural, and social factors (2).

Both anxiety and depression play a central role in the onset and development of AF. Several prospective population-based studies demonstrate that anxiety (7) and depression (8–11) increase the risk of developing AF. Pre-operative anxiety has also been implicated in the onset of AF after coronary artery bypass graft surgery (12). Consistently, cross-sectional population studies demonstrate that the prevalence of AF increases with higher levels of perceived stress (13) and more traumatic life events (14).

Both anxiety and depression are also common consequences of AF. Rates of anxiety and depression reported in AF samples are significantly higher than seen in the general population (15–17). Anxiety has been consistently reported at around 30-35%, while depression rates vary substantially, between 20-45% (15–17). Evidence suggest that AF symptom severity co-occurs with increased levels of anxiety (13, 15, 18) and depression (15, 19, 20).

Given the strong association between AF and mental health, attention needs to be given to managing psychological wellbeing in AF patients. The 2020 European Society of Cardiology (ESC) Guidelines for the Diagnosis and Management of Atrial Fibrillation (21) highlight the importance of measuring the psychological consequences of AF such as anxiety, depression and quality of life. Consistently, an international consortium of AF patients and healthcare professionals has identified patient reported outcomes such as quality of life and emotional functioning as important measures in AF (22). Importantly, evidence from cross-sectional and longitudinal studies highlight that the associations between incident AF and poor psychosocial outcomes are particularly evident for women (16, 23–25).

Psychosocial outcomes are also dependent upon patients’ level of disease-specific knowledge. Evidence suggests that greater knowledge of the disease and its management can reduce uncertainty and alleviate anxiety (26). Patients’ lack of knowledge about AF causes, symptoms and treatments, particularly at the time of diagnosis when the majority of treatment decisions are discussed and made (21), has been identified as a key barrier to therapy uptake and adherence (27, 28). In a study on the educational needs of people living with AF, clinicians indicated that patients’ lack of understanding of AF treatments can contribute to anxiety, stress, and worry about the long‐term impacts of medication management (29). While one study has shown that women with AF have less knowledge than men about AF detection, treatment and impacts (30), another found that women have better symptom knowledge than men (31).

Coping style has also been shown to influence psychological wellbeing. Adaptive coping is directed at managing or altering the problem causing the distress (problem-focused coping) whereas maladapting coping is directed at regulating the emotional response to the problem (emotion-focused coping) or avoiding the problem (avoidant coping) (32, 33). In studies of people with diabetes, cancer and endometriosis, greater engagement in maladaptive coping is associated with higher anxiety and depression, and poorer quality of life, whereas greater engagement in adaptive coping is associated with positive psychological outcomes (34–37).

Thus, both AF knowledge and coping style are likely to have a significant impact on how AF patients experience anxiety and process behavioural responses, which may ultimately affect their quality of life. However, the mediational effects of coping style on the relationship between AF knowledge and psychosocial outcomes in women with AF remain unknown. Thus, the present study aimed to investigate the role of coping style in mediating the relationship between AF knowledge and anxiety, depression and life satisfaction in women with AF.

The specific hypotheses for this study were:

a) There will be significant associations between level of AF knowledge and the three psychosocial outcomes (negative for anxiety and depression, positive for life satisfaction).
b) Adaptive coping (problem-solving coping style) will be directly associated with the three psychosocial outcomes (negative for anxiety and depression, positive for life satisfaction).
c) Maladaptive coping (emotion-focused and avoidant coping styles) will also be directly associated with the three psychosocial outcomes, but in the opposite direction (positive for anxiety and depression, negative for life satisfaction).
d) Given that coping style may interact with level of knowledge and may also be directly associated with the outcomes, there will be significant indirect effects of the three coping styles mediating the relationship between AF knowledge and the three psychosocial outcomes.

## Methods

### Participants and procedures

This study was granted ethical approval through Federation University Research Ethics Committee (approval A21-056). Participants were recruited through advertisements on social media posts and forums, such as Facebook, Reddit, Survey Circle, and Instagram. These advertisements invited potential participants to take part in a confidential and anonymous online survey hosted on Qualtrics that could be accessed through the poster’s hyperlink, weblink and QR code. Inclusion criteria stated that participants were required to be over 18 years of age, female, and Australian residents.

### Measures

The online questionnaire comprised sociodemographic questions, psychosocial measures (anxiety depression, and life satisfaction), self-rated knowledge of AF and coping style.

#### Sociodemographic variables

Participants were asked to indicate their age, marital/partner status and education level, as well as weight (kg) and height (m) which was used to calculate Body Mass Index (BMI=kg/m2)).

#### Anxiety and depression

Anxiety and depression symptoms were assessed using the Hospital Anxiety and Depression rating scale (HADS) (38). The HADS measures symptoms of anxiety (HADS-A; 7 items) and depression (HADS-D; 7 items). Items are rated on a 4-point (0–3) scale with higher scores indicating higher levels of symptoms. Scores range from 0 to 21 and are categorized as normal (0–7), mild (8–10), moderate (11–14), or severe (15–21), with scores >7 indicative of clinically-significant symptoms. The HADS has shown good internal consistency and good concurrent, criterion, and factorial validity with CVD patients (39) and has been used previously with atrial fibrillation patients (40, 41).

#### Life satisfaction

The Satisfaction With Life Scale (SWLS) scale is a 5-item self-report questionnaire scored on a 35-point scale to measure sense of life satisfaction (42, 43). The SWLS has good internal consistency and excellent concurrent validity with other measures of wellbeing (44) and has been previously used to assess life satisfaction in CVD patients (45, 46).

#### Knowledge of atrial fibrillation

Self-rated knowledge of AF was assessed using the following question specifically developed for this study: *“The following question is to get an understanding of how much you know about your condition. Let us know where you place yourself based on your knowledge on Atrial Fibrillation. How well is your understanding of Atrial Fibrillation?”* Responses were measured on a Likert-type scale ranging from 1 (no understanding) through to 5 (Excellent understanding).

#### Coping style

Coping style was assessed using the Brief COPE scale which comprises 28 items rated on a four-point Likert scale, ranging from 1 = I usually don’t do this at all, to 4 = I usually do this a lot. A total of 13 coping strategies are categorized into three main coping styles: problem-focused coping (active coping, instrumental support, positive reframing, and planning), emotion-focused coping (emotional support, venting, humour, acceptance, religion, and self-blame), and avoidant coping (self-distraction, denial, substance use, and behavioural disengagement) (47). Good internal consistency for the subscales have been reported in patients following an acute coronary syndrome (48) with the scale having been used previously with AF patients (49).

### Data analysis

**Figure 1:**
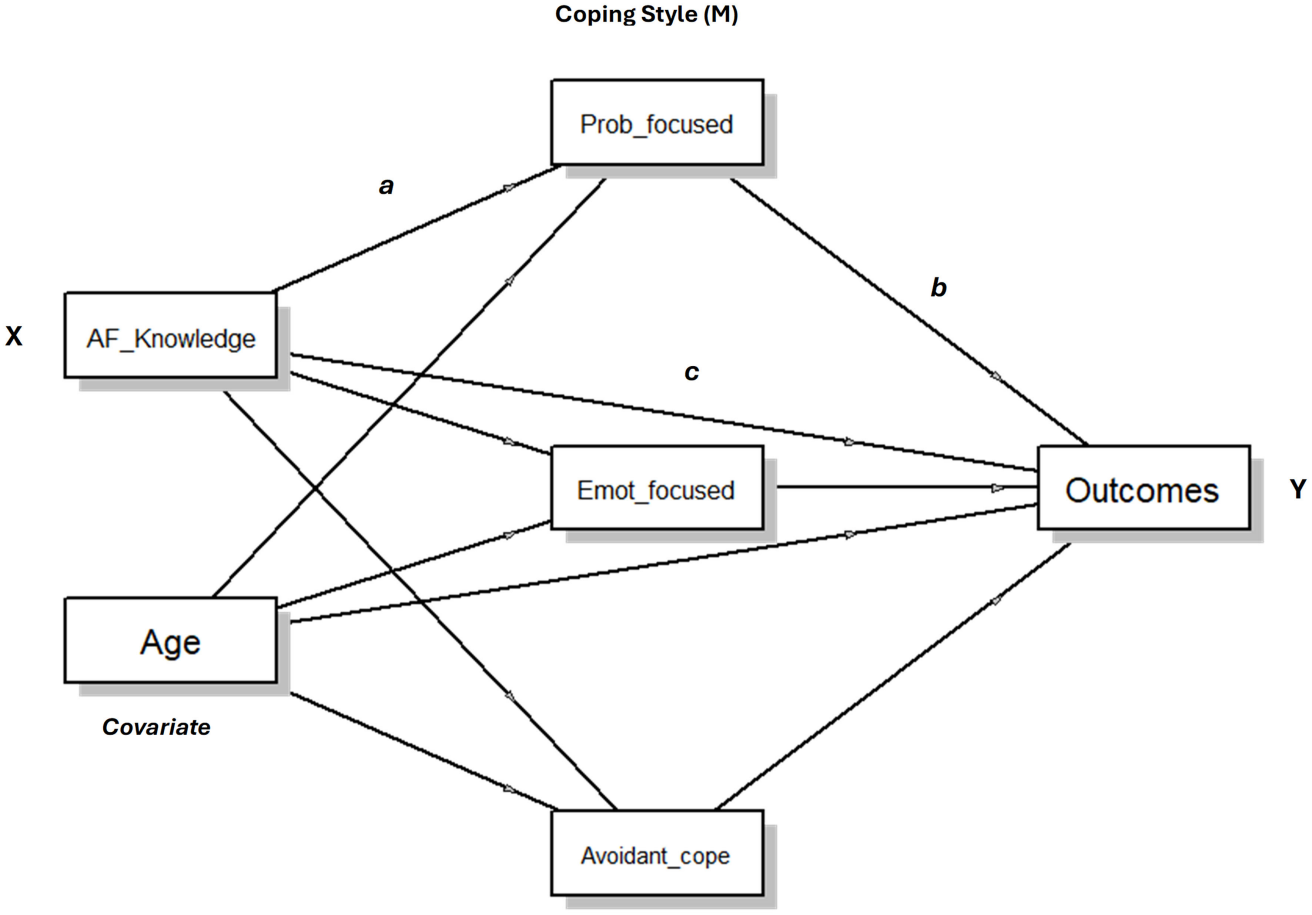
Conceptual model for the process mediation analyses.

After standardization, all variables were assessed for normality and kurtosis. Correlational analyses were undertaken to compare the relationship between standardized study variables. Three parallel process mediation models were run respectively using the outcomes SWLS, HADS-A and HADS-D continuous total scores (Y) (see **Figure 1**). The independent variable for each model was knowledge of AF (X) with age (years) included as a covariate. The proposed mediational variables (M) were the three main styles of coping (problem-focused, emotion-focused and avoidant coping). The proposed direct (c) and indirect effects (ab=a * b) were examined by estimating bias-corrected standardized regression coefficients (betas) using bootstrap analysis (5,000 bootstrap samples) as suggested by Biesanz, Falk, and Savalei (50). Since obesity is associated with occurrence of both atrial fibrillation (51–54) and poorer psychosocial indicators (55–57)a sensitivity analysis, was conducted for all three models with the addition of BMI as a covariate in addition to age. Since emotion-focused and avoidant coping correlated highly, analyses were repeated excluding emotion-focused coping and again excluding avoidant coping. For simplicity and ease of interpretation, these results are not presented as the main analysis. All mediation analyses were conducted using the jAMM module (58) for Jamovi (v2.3) (59).

## Results

A total of 188 females, with ages ranging from 18 to 83 years (mean 48.7, sd 15.5 years), completed the online questionnaire. Sociodemographic characteristics are presented in **Table 1**. Most participants had post-secondary education, two-thirds were married or living with a partner. Over half the participants were overweight or obese (BMI≥25). Only 40% of the sample rated their AF knowledge as good or excellent. Only a quarter of the sample rated their satisfaction with life as satisfied; 79.8% of the sample scored above the clinical HADS cutoff (HADS-A>7) for at least mild anxiety and 70.2% were above the cutoff (HADS-D>7) for at least mild depression.

**Table 1:** Characteristics of participants (n=188).

Characteristic	N (%)
Age group (years)	
18-34	43 (23%)
35-49	53 (28%)
50-64	61 (32%)
≥65	31 (17%)
Education	
Primary	9 (5%)
Secondary	35 (19%)
Trade or TAFE qualification	41 (22%)
University diploma/degree/post-graduate	103 (55%)
Marital status	
Never married	40 (21%)
Widowed	5 (3%)
Divorced or separated	17 (9%)
Married or living with partner	126 (67%)
Body Mass Index (kg/m2)	
Underweight (<18.50)	5 (3%)
Healthy weight (18.5-24.9)	74 (39%)
Overweight (25.0-29.9)	44 (23%)
Obese (≥ 30.0)	65 (35%)
Atrial fibrillation knowledge	
Little or none	45 (23.9)
Moderate	67 (35.6)
Good or Excellent	76 (40.4)
Satisfaction With Life Scale	M 14.2 (SD 9.1)
HADS Anxiety	M 12.9 (SD 7.4)
HADS Depression	M 14.2 (SD 9.1)

After standardization, all variables used for the mediation analyses were assessed for normality and kurtosis and results fell within satisfactory ranges for subsequent statistical analysis. The relationship between the standardized variables is presented in **Table 2**. Correlation between the three major coping styles ranged from *r*=0.31 (problem-focused and avoidant coping) through to *r*=.71 (emotion-focused and avoidant coping). AF knowledge was moderately negatively correlated with anxiety (*r* =-.50, *p* <.001) and depression (*r* =-.53, *p* <.001) and moderately positively correlated with life satisfaction (*r*=.40, *p* <.001). Emotion focused coping and avoidant coping styles were significantly positively correlated with both anxiety and depression, and negatively correlated with life satisfaction. Age correlated positively with life satisfaction (*r*=.41, *p* <.001) and negatively with anxiety (*r*= -.47, *p* <.001) and depression scores (*r*= -.45, *p* <.001). Since age correlated significantly with most variables it was included as a covariate in the mediation analyses. BMI was significantly negatively correlated with emotion focused coping and avoidant coping styles, and life satisfaction and positively correlated with both anxiety and depression.

**Table 2:** Relationship between study variables. .

		Age	BMI	AFK	PC	EC	AC	SWLS	HADSA
Age	Pearson’s r	—							
	p-value	—							
BMI	Pearson’s r	0.269	—						
	p-value	< .001	—						
AFK	Pearson’s r	0.372	0.300	—					
	p-value	< .001	< .001	—					
PC	Pearson’s r	0.068	0.069	0.045	—				
	p-value	0.355	0.344	0.544	—				
EC	Pearson’s r	-0.291	-0.297	-0.228	0.629	—			
	p-value	< .001	< .001	0.002	< .001	—			
AC	Pearson’s r	-0.345	-0.452	-0.398	0.313	0.705	—		
	p-value	< .001	< .001	< .001	< .001	< .001	—		
SWLS	Pearson’s r	0.409	0.408	0.401	0.100	-0.292	-0.498	—	
	p-value	< .001	< .001	< .001	0.174	< .001	< .001	—	
HADS-A	Pearson’s r	-0.472	-0.533	-0.502	-0.029	0.427	0.650	-0.748	—
	p-value	< .001	< .001	< .001	0.694	< .001	< .001	< .001	—
HADS-D	Pearson’s r	-0.453	-0.547	-0.530	-0.035	0.468	0.640	-0.768	0.873
	p-value	< .001	< .001	< .001	0.632	< .001	< .001	< .001	< .001

BMI, Body Mass Index; AFK, atrial fibrillation knowledge; PC, problem focused coping style, EC, emotion focused coping style, AC, avoidant coping style; SWLS, Satisfaction With Life Scale; HADS-A, Hospital Anxiety and Depression Scale – Anxiety; HADS-D, Hospital Anxiety and Depression Scale – Depression

### Mediation analyses

Three mediation models with standardized variables were tested (i.e., Model A: AF Knowledge → Coping style → life satisfaction; Model B: AF Knowledge → Coping style → Anxiety; Model C: AF Knowledge → Coping style → Depression) with age (years) as a covariate in all analyses.

#### Model A: knowledge of atrial fibrillation and life satisfaction

**Figure 2:**
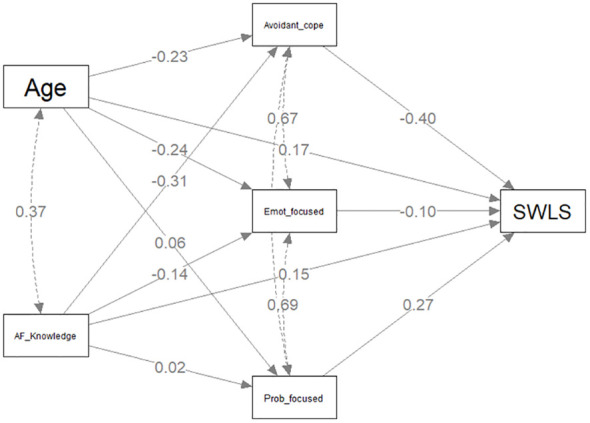
Mediation model predicting Satisfaction With Life Scale scores.

The individual regressions for direct and indirect components predicting total SWLS scores are presented in **Table 3** with the statistical model presented in **Figure 2**. The first two requirements for the mediation procedure are that the independent variable (AF Knowledge) be related to the dependent variable (SWLS) and the mediator (three coping styles). Satisfying this requirement, there was a direct significant positive relationship between AF Knowledge and SWLS scores (*β*= .146, *p* =.033) and a significant negative relationship between AF Knowledge and avoidance coping style (*β* = -.313, *p* <.001). This relationship between avoidant coping style and AF knowledge is common to all three models. The indirect relationship (AF Knowledge → Avoidant Coping style → life satisfaction) was significant (β =.124, p <.001). Thus, with three coping styles competing together, significant partial mediation effects were observed for avoidant coping style, but not for problem-focused or emotion-focused coping. There was, however, a direct positive relationship between problem-focused coping and higher SWLS scores (*β*= .266, *p* =.007) and a direct negative relationship between avoidant coping and SWLS (*β*= -.397, *p* <.001). The covariate age was positively related to higher SWLS scores (*β*= .172, *p* = .026).

**Table 3:** Associations between knowledge of atrial fibrillation and Satisfaction With Life Scale total scores mediated by three styles of coping.

Effect	Estimate	SE	Lower	Upper	β	z	*p*
Indirect							
AFK ⇒ PC ⇒ SWLS	0.069	0.244	-0.344	0.678	0.006	0.284	0.777
AFK ⇒ EC ⇒ SWLS	0.155	0.242	-0.207	0.783	0.013	0.641	0.522
AFK ⇒ AC⇒ SWLS	1.439	0.421	0.724	2.402	0.124	3.414	**< .001**
Age ⇒ PC ⇒ SWLS	0.009	0.014	-0.015	0.045	0.016	0.650	0.516
Age ⇒ EC ⇒ SWLS	0.014	0.020	-0.021	0.061	0.023	0.677	0.498
Age ⇒ AC⇒ SWLS	0.053	0.021	0.019	0.103	0.090	2.487	**0.013**
Component							
AFK ⇒ PC	0.125	0.411	-0.660	0.963	0.022	0.305	0.761
PC ⇒ SWLS	0.552	0.203	0.136	0.947	0.266	2.720	0.007
AFK ⇒ EC	-1.185	0.642	-2.430	0.089	-0.139	-1.844	0.065
EC ⇒ SWLS	-0.131	0.176	-0.451	0.238	-0.096	-0.745	0.457
AFK ⇒ AC	-1.889	0.451	-2.711	-0.946	-0.313	-4.186	**< .001**
AC⇒ SWLS	-0.761	0.145	-1.040	-0.476	-0.397	-5.271	**< .001**
Age ⇒ PC	0.017	0.024	-0.031	0.063	0.059	0.712	0.477
Age ⇒ EC	-0.103	0.036	-0.173	-0.033	-0.239	-2.905	**0.004**
Age ⇒ AC	-0.070	0.024	-0.117	-0.024	-0.228	-2.963	**0.003**
Direct							
AFK ⇒ SWLS	1.685	0.791	0.148	3.238	0.146	2.132	**0.033**
Age ⇒ SWLS	0.101	0.045	0.016	0.194	0.172	2.232	**0.026**
Total							
AFK ⇒ SWLS	3.348	0.796	1.789	4.908	0.289	4.208	**< .001**
Age ⇒ SWLS	0.177	0.041	0.098	0.257	0.301	4.379	**< .001**

N=188; AFK, atrial fibrillation knowledge; PC, problem focused coping style, EC, emotion focused coping style, AC, avoidant coping style; SWLS, Satisfaction With Life Scale total score; significant associations in bold.

#### Model B: knowledge of atrial fibrillation and anxiety

**Figure 3:**
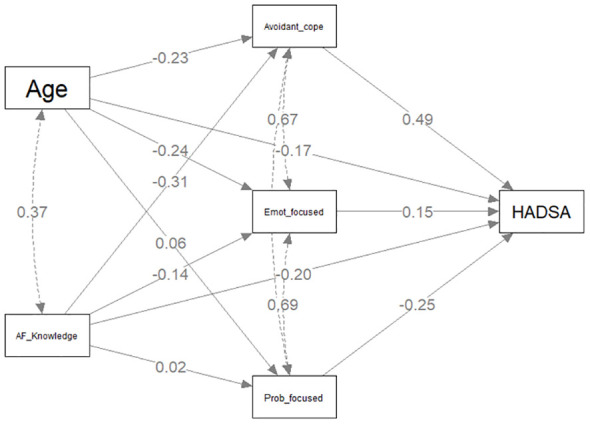
Mediation model predicting HADS Anxiety scores.

The individual regressions for direct and indirect components predicting total HADS-A scores are presented in **Table 4** with the statistical model presented in **Figure 3**. There was a direct significant negative relationship between AF Knowledge and HADS-A scores (*β*= -.200, *p* <.001). Of the three coping styles, only avoidant coping style was significantly associated with AF knowledge (*β* =-0.313, *p* <.001) and partial mediation was confirmed with the indirect effect (*β*= -0.152, *p* <.001). Both problem-focused coping (*β*=-.255, *p* <.001) and avoidant coping styles (*β*= .487, *p* <.001) had direct associations with HADS-A scores. Age was negatively associated with HADS-A scores (*β*= -.169, *p* =.004).

**Table 4:** Associations between knowledge of atrial fibrillation and HADS Anxiety total scores mediated by three styles of coping.

Effect	Estimate	SE	Lower	Upper	β	z	*p*
Indirect							
AFK ⇒ PC ⇒ HADSA	-0.047	0.166	-0.384	0.291	-0.006	-0.285	0.776
AFK ⇒ EC ⇒ HADSA	-0.171	0.156	-0.659	0.015	-0.021	-1.098	0.272
AFK ⇒ AC⇒ HADSA	-1.256	0.369	-2.115	-0.640	-0.152	-3.407	**< .001**
Age ⇒ PC ⇒ HADSA	-0.006	0.009	-0.028	0.010	-0.015	-0.677	0.498
Age ⇒ EC ⇒ HADSA	-0.015	0.012	-0.047	0.001	-0.036	-1.272	0.203
Age ⇒ AC⇒ HADSA	-0.046	0.017	-0.084	-0.018	-0.111	-2.740	**0.006**
Component							
AFK ⇒ PC	0.125	0.420	-0.729	0.913	0.022	0.299	0.765
PC ⇒ HADSA	-0.376	0.110	-0.603	-0.169	-0.255	-3.412	**< .001**
AFK ⇒ EC	-1.185	0.646	-2.432	0.037	-0.139	-1.833	0.067
EC ⇒ HADSA	0.144	0.096	-0.028	0.352	0.149	1.506	0.132
AFK ⇒ AC	-1.889	0.445	-2.728	-0.993	-0.313	-4.249	**< .001**
AC⇒ HADSA	0.665	0.107	0.450	0.863	0.487	6.221	**< .001**
Age ⇒ PC	0.017	0.023	-0.028	0.064	0.059	0.718	0.473
Age ⇒ EC	-0.103	0.035	-0.171	-0.033	-0.239	-2.957	**0.003**
Age ⇒ AC	-0.070	0.023	-0.117	-0.025	-0.228	-3.000	**0.003**
Direct							
AFK ⇒ HADSA	-1.651	0.442	-2.554	-0.802	-0.200	-3.735	**< .001**
Age ⇒ HADSA	-0.071	0.025	-0.118	-0.020	-0.169	-2.875	**0.004**
Total							
AFK ⇒ HADSA	-3.125	0.525	-4.153	-2.097	-0.379	-5.957	**< .001**
Age ⇒ HADSA	-0.139	0.027	-0.191	-0.086	-0.331	-5.196	**< .001**

N=188; AFK, atrial fibrillation knowledge; PC, problem focused coping style, EC, emotion focused coping style, AC, avoidant coping style; HADSA, Hospital Anxiety and Depressions Anxiety Scale total score; significant associations in bold.

#### Model C: knowledge of atrial fibrillation and depression

**Figure 4:**
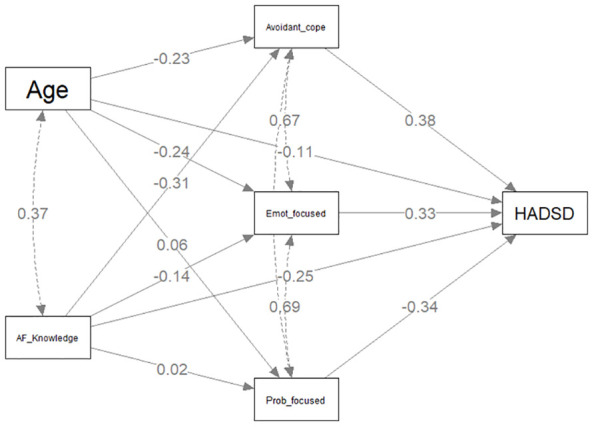
Mediation model predicting HADS Depression scores.

The individual regressions for direct and indirect components predicting total HADS-D scores are presented in **Table 5** with the statistical model presented in **Figure 4**. AF knowledge was directly negatively associated with HADS-D total scores (*β*= -.247, *p* <.001). As was the case with models A and B, only the partial mediation effect for avoidant coping style on the outcome variable was confirmed with a significant indirect effect (*β*= -.119, *p* <.001). In contrast to models A and B, all three styles of coping had direct significant effects on HADS-D scores (Problem-focused *β*= -.340, *p* <.001; Emotion-focused *β*= .325, *p* <.001; Avoidant coping *β*= .380, *p* <.001). Age was significantly negatively related to HADS-D scores (*β*= -.113, *p* =.044).

**Table 5:** Associations between knowledge of atrial fibrillation and HADS Depression total scores mediated by three styles of coping.

Effect	Estimate	SE	Lower	Upper	β	z	*p*
Indirect							
AFK ⇒ PC ⇒ HADSD	-0.072	0.247	-0.576	0.405	-0.008	-0.290	0.772
AFK ⇒ EC ⇒ HADSD	-0.425	0.256	-1.033	-0.008	-0.045	-1.657	0.097
AFK ⇒ AC⇒ HADSD	-1.118	0.328	-1.869	-0.558	-0.119	-3.410	**< .001**
Age ⇒ PC ⇒ HADSD	-0.010	0.014	-0.037	0.017	-0.020	-0.709	0.478
Age ⇒ EC ⇒ HADSD	-0.037	0.016	-0.078	-0.012	-0.078	-2.281	**0.023**
Age ⇒ AC⇒ HADSD	-0.041	0.016	-0.075	-0.014	-0.087	-2.655	**0.008**
Component							
AFK ⇒ PC	0.125	0.418	-0.681	0.960	0.022	0.299	0.765
PC ⇒ HADSD	-0.572	0.110	-0.801	-0.367	-0.340	-5.198	**< .001**
AFK ⇒ EC	-1.185	0.641	-2.433	0.088	-0.139	-1.848	0.065
EC ⇒ HADSD	0.359	0.092	0.179	0.537	0.325	3.922	**< .001**
AFK ⇒ AC	-1.889	0.447	-2.764	-1.025	-0.313	-4.229	**< .001**
AC⇒ HADSD	0.591	0.104	0.373	0.786	0.380	5.679	**< .001**
Age ⇒ PC	0.017	0.023	-0.029	0.062	0.059	0.730	0.465
Age ⇒ EC	-0.103	0.035	-0.171	-0.034	-0.239	-2.963	**0.003**
Age ⇒ AC	-0.070	0.024	-0.115	-0.024	-0.228	-2.977	**0.003**
Direct							
AFK ⇒ HADSD	-2.317	0.449	-3.248	-1.475	-0.247	-5.159	**< .001**
Age ⇒ HADSD	-0.054	0.027	-0.107	-2.70	-0.113	-2.010	**0.044**
Total							
AFK ⇒ HADSD	-3.931	0.593	-5.092	-2.770	-0.419	-6.635	**< .001**
Age ⇒ HADSD	-0.142	0.030	-0.201	-0.083	-0.297	-4.705	**< .001**

N=188; AFK, atrial fibrillation knowledge; PC, problem focused coping style, EC, emotion focused coping style, AC, avoidant coping style; HADSD, Hospital Anxiety and Depressions Depression Scale total score; significant associations in bold.

### Sensitivity analysis

Analysis of all three models were repeated with the addition of BMI (kg/m2) as an additional covariate. BMI was negatively associated with HADS-A (*β*= -.201, *p* <.001) and HADS-D (*β*= -.209, *p* <.001) but was not significantly associated with SWLS score (*β*= .128, *p* =.056). The partial mediation effects observed in the previous models were weakened but significance maintained with significant indirect effects only for avoidant coping style in all three sensitivity models (SWLS *β*= .083, *p* =.009; HADS-A *β*= -.098, *p* =.003; HADS-D *β*= -.072, *p* =.007). Since avoidant coping and emotion-focused coping correlated highly (*r*=0.7), the analysis of all models was repeated excluding emotion-focused coping and again excluding avoidant coping. The key results (significant partial mediating effect of avoidant coping) did not change when emotion-focused coping was removed from all three models. When avoidant coping was removed from all three models, no partial mediation effect was observed for either problem-focused coping or emotion-focused coping, regardless of whether BMI was included or excluded as a covariate. With exclusion of avoidant coping from the models, the direct relationship between emotion-focused coping and the three outcomes also became significant.

The indirect effect of avoidant coping on psychosocial outcomes was also examined for a subset of participants who had good or excellent AF knowledge (**Table 6**). Participants who scored above the median on avoidant coping performed significantly worse on all three psychosocial outcomes than those who scored low on avoidant coping (SWLS F=10.9, df(1,74), *p* =.002; HADS-A F=15.2, df(1,74) *p* =<.001; HADS-D F=19.6, df(1,74) *p* <.001). By contrast, for problem focused coping, which did not have a significant indirect effect on outcomes, there were no significant differences between those who scored low versus high.

**Table 6:** Effect of low versus high avoidant coping and problem focused coping on psychosocial outcomes for subset of participants with good or excellent atrial fibrillation knowledge.

	Satisfaction With Life		HADS Anxiety		HADS Depression	
	Mean	SD	Mean	SD	Mean	SD
Low avoidant coping (n=52)	20.1	8.2	9.1	5.4	6.9	5.5
High avoidant coping (n=24)	13.5	8.1	14.5	6.0	13.5	6.9
Significance	F=10.9, df _1,74_, *p* =.002		F=15.2, df _1,74_ *p* =<.001		F=19.6, df _1,74_ *p* <.001	
Low problem focused coping (n=35)	16.3	9.1	11.3	6.2	9.5	7.1
High problem focused coping (n=38)	19.6	8.2	10.6	6.1	8.7	6.6
Significance	F=2.64, df _1,71_, *p* =.109		F=0.24, df _1,71_. P =.627		F=0.24, df _1,71_, *p*=.629	

## Discussion

The present study investigated the relationships between AF knowledge, coping and psychological outcomes. To our knowledge, these findings are the first to quantitatively demonstrate moderate positive associations of AF knowledge with life satisfaction and moderate negative associations of AF knowledge with anxiety and depression. These findings are consistent with those of previous qualitative work (29) and also with previous findings that demonstrate inadequate knowledge is associated with uncertainty and stress (26).

Problem-solving coping style was also directly associated with the three psychosocial outcomes, being negative for anxiety and depression, and positive for life satisfaction. In contrast, the maladaptive emotion-focused and avoidant coping styles were directly associated with higher anxiety and depression and lower life satisfaction. These findings are consistent with those from studies of patients with various chronic conditions that have found poorer psychosocial outcomes in patients with maladaptive coping styles compared to better psychosocial outcomes with positive or adaptive coping styles (34–37). Given the cross-sectional design of our study, we are unable to eliminate the possibility that the presence of depressive symptoms may have influenced maladaptive coping styles, specifically avoidant coping. This notion aligns with findings from a prior study conducted by Trivedi and colleagues in stable heart failure patients (60). Their research revealed significant associations between depressive symptoms and avoidant coping, low perceived social support, and pessimism. Notably, it’s worth mentioning that in their study, the majority of participants were male (67%), who often report lower depression rates than females (61).

Importantly, the current study provided support for a crucial role of coping styles in mediating the association between AF knowledge and psychosocial outcomes. Mediation analyses revealed that avoidant coping style partially mediated the relationship between AF knowledge and psychosocial outcomes. Specifically, when avoidant coping is low, there is the expected positive linear relationship between AF knowledge and life satisfaction scores. When avoidant coping is high, this linear association is significantly diminished and overall satisfaction with life is lower. When avoidant coping is high, AF knowledge is less influential on lowering anxiety or depression scores. Coping style, therefore, appears to have a crucial impact on how patients experience anxiety and process their behavioural response (32, 33).

The present findings have important clinical implications in terms of the development and trailing of interventions to improve AF knowledge as a means of improving psychological outcomes in AF patients. Previous studies have shown improved quality of life in AF patients who were supported to improve their knowledge of their condition (62–64). In addition to patient education, the findings of this study also highlight the potential importance of problem-solving coping strategies in improving psychosocial outcomes in patients with AF. Effective patient education has the potential to improve patients’ understanding of their condition and its management, leading to improved coping strategies and better psychosocial outcomes. Adaptive problem-solving coping strategies in particular can help patients manage the problem causing their distress and reduce anxiety and depression, while enhancing satisfaction with life (65, 66). In contrast, maladaptive coping has been associated with poorer psychosocial outcomes in other patient groups such as heart failure patients (60) and has the potential to negate the potentially beneficial effects of patient knowledge.

In relation to both adaptive and maladaptive coping, psychotherapy offers a structured and supportive environment for patients to explore and develop coping strategies that can enhance their ability to manage the emotional challenges associated with AF (67). By addressing issues such as anxiety, depression, and the fear of sudden cardiac events, psychotherapeutic approaches like cognitive-behavioural therapy (CBT), relaxation techniques, and stress management can equip patients with the tools to better navigate the psychological impact of their condition (68–70). Further, randomised trials have shown that interventions aimed at improving patient education and knowledge about AF result in enhanced patient adherence, decreased treatment-related complications (71) and enhanced quality of life (72, 73). This finding is consistent with research on other health conditions on the impact of knowledge on anxiety and stress levels (74–76).

### Limitations

In common with cross-sectional online surveys such as this, the measures of anxiety, depression, satisfaction with life and knowledge of AF all relied on self-report, with no objective measure of these. This study relied on self-reported perceived knowledge of AF and did not use one of the several validated instruments available to assess AF knowledge (77). In addition, there was no measure of AF symptom severity. Our patients were all women and were sourced via social media and reported higher levels of anxiety and depression than would be expected from hospital sourced samples (78). Since our analyses involved continuous scale scores instead of clinical categories of anxiety and depression, it is unlikely that the mediation analysis would have been adversely affected by this limitation. This study did not explore the potential influence of potential confounders, such as socioeconomic status, medication use or comorbidities, on the relationship between AF knowledge and psychosocial outcomes. Given the self-selected nature of the sample, the findings should be interpreted cautiously until they can be replicated in a consecutive representative sample of confirmed diagnosed AF patients.

## Conclusions

It is notable that current Atrial Fibrillation Guidelines (3, 21, 79, 80) provide scant or no attention whatsoever to the psychological aspects of living with AF. The results of the current study add to a growing body of literature that highlights the psychological challenges of living with AF and indicate that such issues could be incorporated into comprehensive management guidelines. More specifically, these findings highlight the need for adequate psychological screening of cardiac patients as well as effective psychotherapeutic interventions to empower patients to better understand and cope with the emotional and psychological aspects of their condition.

## Data availability statement

The raw data supporting the conclusions of this article will be made available by the authors, without undue reservation.

## Ethics statement

The studies involving humans were approved by Federation University Ethics Committee. The studies were conducted in accordance with the local legislation and institutional requirements. The participants provided their written informed consent to participate in this study.

## Author contributions

MG: Data curation, Formal Analysis, Writing – original draft. MS: Investigation, Writing – review & editing. LS: Formal Analysis, Writing – review & editing. BM: Supervision, Writing – review & editing. AJ: Formal Analysis, Methodology, Writing – original draft. MA: Conceptualization, Funding acquisition, Investigation, Project administration, Writing – original draft.

## References

[1] January CT Wann LS Alpert JS Calkins H Cigarroa JE Cleveland JC Jr. 2014 AHA/ACC/HRS guideline for the management of patients with atrial fibrillation: a report of the American College of Cardiology/American Heart Association Task Force on practice guidelines and the Heart Rhythm Society. Circulation. (2014) 130:e199–267. doi: 10.1161/cir.0000000000000041 PMC4676081 24682347

[2] Matei LL Siliste C Vinereanu D. Modifiable risk factors and atrial fibrillation: the quest for a personalized approach. Maedica (Bucur). (2021) 16:88–96. doi: 10.26574/maedica.2020.16.1.88 34221161 PMC8224718

[3] Brieger D Amerena J Attia J Bajorek B Chan KH Connell C. National heart foundation of Australia and the cardiac society of Australia and New Zealand: Australian clinical guidelines for the diagnosis and management of atrial fibrillation 2018. Heart Lung Circulation. (2018) 27:1209–66. doi: 10.1016/j.hlc.2018.06.1043 30077228

[4] Rolfes CD Howard SA Goff RP Iaizzo PA. Cardiac remodeling as a consequence of atrial fibrillation: An anatomical study of perfusion-fixed human heart specimens. J Geriatr Cardiol. (2011) 8:141–6. doi: 10.3724/sp.J.1263.2011.00141 PMC3390070 22783300

[5] Ball J Thompson DR Ski CF Carrington MJ Gerber T Stewart S. Estimating the current and future prevalence of atrial fibrillation in the Australian adult population. Article. Med J Australia. (2015) 202:32–6. doi: 10.5694/mja14.00238 25588442

[6] Zoni-Berisso M Lercari F Carazza T Domenicucci S. Epidemiology of atrial fibrillation: European perspective. Clin Epidemiol. (2014) 6:213–20. doi: 10.2147/clep.S47385 PMC4064952 24966695

[7] Cheng YF Leu HB Su CC Huang CC Chiang CH Huang PH. Association between panic disorder and risk of atrial fibrillation:a nationwide study. Psychosom Med. (2013) 75:30–5. doi: 10.1097/PSY.0b013e318273393a 23107841

[8] Garg PK Claxton JS Soliman EZ Chen LY Lewis TT Mosley T. Associations of anger, vital exhaustion, anti-depressant use, and poor social ties with incident atrial fibrillation: The Atherosclerosis Risk in Communities Study. Eur J Prev Cardiol. (2021) 28:633–40. doi: 10.1177/2047487319897163 PMC8981832 34021575

[9] Garg PK O'Neal WT Diez-Roux AV Alonso A Soliman EZ Heckbert S. Negative affect and risk of atrial fibrillation: MESA. J Am Heart Assoc. (2019) 8:e010603. doi: 10.1161/jaha.118.010603 30563392 PMC6405728

[10] Fenger-Grøn M Vestergaard M Pedersen HS Frost L Parner ET Ribe AR. Depression, antidepressants, and the risk of non-valvular atrial fibrillation: A nationwide Danish matched cohort study. Eur J Prev Cardiol. (2019) 26:187–95. doi: 10.1177/2047487318811184 30452291

[11] Egeberg A Khalid U Gislason GH Mallbris L Skov L Hansen PR. Association between depression and risk of atrial fibrillation and stroke in patients with psoriasis: a Danish nationwide cohort study. Br J Dermatol. (2015) 173:471–9. doi: 10.1111/bjd.13778 25781210

[12] Alkan Kayhan S Güner E Hanedan MO Topal Çolak E Mataraci İ. Relationship between preoperative anxiety and atrial fibrillation after coronary artery bypass graft surgery. J Nurs Res. (2022) 30:e187. doi: 10.1097/jnr.0000000000000473 35050953

[13] O'Neal WT Qureshi W Judd SE Glasser SP Ghazi L Pulley L. Perceived stress and atrial fibrillation: the REasons for geographic and racial differences in stroke study. Ann Behav Med. (2015) 49:802–8. doi: 10.1007/s12160-015-9715-2 PMC4936185 26044964

[14] Westcott SK Beach LY Matsushita F Albert CM Chatterjee N Wong J. Relationship between psychosocial stressors and atrial fibrillation in women >45 years of age. Am J Cardiol. (2018) 122:1684–7. doi: 10.1016/j.amjcard.2018.07.044 PMC6242716 30266256

[15] Koleck TA Mitha SA Biviano A Caceres BA Corwin EJ Goldenthal I. Exploring Depressive Symptoms and Anxiety Among Patients With Atrial Fibrillation and/or Flutter at the Time of Cardioversion or Ablation. J Cardiovasc Nurs. (2021) 36:470–81. doi: 10.1097/jcn.0000000000000723 PMC9126094 32675627

[16] Polikandrioti M Koutelekos I Vasilopoulos G Gerogianni G Gourni M Zyga S. Anxiety and depression in patients with permanent atrial fibrillation: prevalence and associated factors. Cardiol Res Pract. (2018) 2018:7408129. doi: 10.1155/2018/7408129 29670767 PMC5836417

[17] Bostrom JA Saczynski JS Hajduk A Donahue K Rosenthal LS Browning C. Burden of psychosocial and cognitive impairment in patients with atrial fibrillation. Crit Pathw Cardiol. (2017) 16:71–5. doi: 10.1097/hpc.0000000000000101 PMC5434700 28509707

[18] Thompson TS Barksdale DJ Sears SF Mounsey JP Pursell I Gehi AK. The effect of anxiety and depression on symptoms attributed to atrial fibrillation. Pacing Clin Electrophysiol. (2014) 37:439–46. doi: 10.1111/pace.12292 24215267

[19] Isakade N Tahhan A Sandesara P Hayek S Alkhoder A Gafeer M. ASSOCIATION BETWEEN DEPRESSION AND THE PREVALENCE OF ATRIAL FIBRILLATION. J Am Coll Cardiol. (2019) 73:1885–5. doi: 10.1016/S0735-1097(19)32491-X

[20] Kupper N van den Broek KC Widdershoven J Denollet J. Subjectively reported symptoms in patients with persistent atrial fibrillation and emotional distress. Front Psychol. (2013) 4:192. doi: 10.3389/fpsyg.2013.00192 23630509 PMC3634051

[21] Hindricks G Potpara T Dagres N Arbelo E Bax JJ Blomström-Lundqvist C. 2020 ESC Guidelines for the diagnosis and management of atrial fibrillation developed in collaboration with the European Association for Cardio-Thoracic Surgery (EACTS): The Task Force for the diagnosis and management of atrial fibrillation of the European Society of Cardiology (ESC) Developed with the special contribution of the European Heart Rhythm Association (EHRA) of the ESC. Eur Heart J. (2021) 42:373–498. doi: 10.1093/eurheartj/ehaa612 32860505

[22] Seligman WH Das-Gupta Z Jobi-Odeneye AO Arbelo E Banerjee A Bollmann A. Development of an international standard set of outcome measures for patients with atrial fibrillation: a report of the International Consortium for Health Outcomes Measurement (ICHOM) atrial fibrillation working group. Eur Heart J. (2020) 41:1132–40. doi: 10.1093/eurheartj/ehz871 PMC7060456 31995195

[23] Gleason KT Dennison Himmelfarb CR Ford DE Ford DE Lehmann H Samuel L. Association of sex, age and education level with patient reported outcomes in atrial fibrillation. BMC Cardiovasc Disord. (2019) 19:85. doi: 10.1186/s12872-019-1059-6 30953478 PMC6451250

[24] Dagres N Nieuwlaat R Vardas PE Andresen D Lévy S Cobbe S. Gender-related differences in presentation, treatment, and outcome of patients with atrial fibrillation in europe: A report from the euro heart survey on atrial fibrillation. J Am Coll Cardiol. (2007) 49:572–7. doi: 10.1016/j.jacc.2006.10.047 17276181

[25] Reynolds MR Lavelle T Essebag V Cohen DJ Zimetbaum P. Influence of age, sex, and atrial fibrillation recurrence on quality of life outcomes in a population of patients with new-onset atrial fibrillation: the Fibrillation Registry Assessing Costs, Therapies, Adverse events and Lifestyle (FRACTAL) study. Am Heart J. (2006) 152:1097–103. doi: 10.1016/j.ahj.2006.08.011 PMC1820843 17161061

[26] Patel D Mc Conkey ND Sohaney R Mc Neil A Jedrzejczyk A Armaganijan L. A systematic review of depression and anxiety in patients with atrial fibrillation: the mind-heart link. Cardiovasc Psychiatry Neurol. (2013) 2013:159850. doi: 10.1155/2013/159850 23710335 PMC3655604

[27] Konieczyńska M Bijak P Malinowski KP Undas A. Knowledge about atrial fibrillation and anticoagulation affects the risk of clinical outcomes. Thromb Res. (2022) 213:105–12. doi: 10.1016/j.thromres.2022.03.011 35320762

[28] Salmasi S De Vera MA Barry A Bansback N Harrison M Lynd LD. Assessment of condition and medication knowledge gaps among atrial fibrillation patients: A systematic review and meta-analysis. Ann Pharmacother. (2019) 53:773–85. doi: 10.1177/1060028019835845 30829052

[29] Ferguson C Hickman LD Lombardo L Downie A Bajorek B Ivynian S. Educational needs of people living with atrial fibrillation: A qualitative study. J Am Heart Assoc. (2022) 11:e025293. doi: 10.1161/JAHA.122.025293 35876410 PMC9375481

[30] Koponen L Rekola L Ruotsalainen T Lehto M Leino-Kilpi H Voipio-Pulkki LM. Patient knowledge of atrial fibrillation: 3-month follow-up after an emergency room visit. J Adv Nurs. (2008) 61:51–61. doi: 10.1111/j.1365-2648.2007.04465.x 18173735

[31] Kueh YC Morris T Ismail AA. The effect of diabetes knowledge and attitudes on self-management and quality of life among people with type 2 diabetes. Psychol Health Med. (2017) 22:138–44. doi: 10.1080/13548506.2016.1147055 26851120

[32] Skinner EA Edge K Altman J Sherwood H. Searching for the structure of coping: a review and critique of category systems for classifying ways of coping. Psychol Bull. (2003) 129:216–69. doi: 10.1037/0033-2909.129.2.216 12696840

[33] Lazarus RS Folkman S. Stress, appraisal, and coping. New York: Springer publishing company (1984).

[34] Beesley VL Smith DD Nagle CM Friedlander M Grant P DeFazio A. Coping strategies, trajectories, and their associations with patient-reported outcomes among women with ovarian cancer. Support Care Cancer. (2018) 26:4133–42. doi: 10.1007/s00520-018-4284-0 29948398

[35] Donatti L Ramos DG Andres MP Passman LJ Podgaec S. Patients with endometriosis using positive coping strategies have less depression, stress and pelvic pain. Einstein (Sao Paulo). (2017) 15:65–70. doi: 10.1590/s1679-45082017ao3911 28444092 PMC5433310

[36] Guan T Santacroce SJ Chen DG Song L. Illness uncertainty, coping, and quality of life among patients with prostate cancer. Psychooncology. (2020) 29:1019–25. doi: 10.1002/pon.5372 PMC7440775 32128938

[37] Knowles SR Apputhurai P O'Brien CL Ski CF Thompson DR Castle DJ. Exploring the relationships between illness perceptions, self-efficacy, coping strategies, psychological distress and quality of life in a cohort of adults with diabetes mellitus. Psychol Health Med. (2020) 25:214–28. doi: 10.1080/13548506.2019.1695865 31790287

[38] Zigmond AS Snaith RP. The hospital anxiety and depression scale. Acta Psychiatr Scand. (1983) 67:361–70. doi: 10.1111/j.1600-0447.1983.tb09716.x 6880820

[39] Bond R Burns J Ehrlich-Jones L. Measurement characteristics and clinical utility of the hospital anxiety and depression scale among adults with cardiovascular disease. Arch Phys Med Rehabilitation. (2019) 100:2219–20. doi: 10.1016/j.apmr.2019.07.004

[40] Li P Yu D Yan B. Nurse-led multi-component behavioural activation programme to improve health outcomes in patients with atrial fibrillation: a mixed-methods study and feasibility analysis. Eur J Cardiovasc Nursing. (2022) 22(6):655–63. doi: 10.1093/eurjcn/zvac104 36394495

[41] Uchmanowicz I Lomper K Gros M Kałużna-Oleksy M Jankowska EA Rosińczuk J. Assessment of frailty and occurrence of anxiety and depression in elderly patients with atrial fibrillation. Clin Interventions Aging. (2020) 15:1151–61. doi: 10.2147/CIA.S258634 PMC7371919 32764902

[42] Diener E Emmons RA Larsen RJ Griffin S. The satisfaction with life scale. J Pers Assess. (1985) 49:71–5. doi: 10.1207/s15327752jpa4901_13 16367493

[43] Emerson SD Guhn M Gadermann AM. Measurement invariance of the Satisfaction with Life Scale: Reviewing three decades of research. Qual Life Res. (2017) 26:2251–64. doi: 10.1007/s11136-017-1552-2 28324322

[44] Magyar-Moe JL. Chapter 3 - Positive Psychological Tests and Measures. In: Magyar-Moe JL, editor. Therapist's Guide to Positive Psychological Interventions. San Diego: Academic Press (2009). p. 43–72.

[45] Kang W. Personality traits predict life satisfaction in coronary heart disease (CHD) patients. J Clin Med. (2022) 11(21):6312. doi: 10.3390/jcm11216312 36362545 PMC9654296

[46] Natt och Dag Y Engström G Rosvall M. Life satisfaction and coronary atherosclerosis: The SCAPIS study. J Psychosomatic Res. (2022) 152:110663. doi: 10.1016/j.jpsychores.2021.110663 34798453

[47] Carver CS. You want to measure coping but your protocol's too long: consider the brief COPE. Int J Behav Med. (1997) 4:92–100. doi: 10.1207/s15327558ijbm0401_6 16250744

[48] Chiavarino C Rabellino D Ardito RB Cavallero E Palumbo L Bergerone S. Emotional coping is a better predictor of cardiac prognosis than depression and anxiety. J Psychosomatic Res. (2012) 73:473–5. doi: 10.1016/j.jpsychores.2012.10.002 23148818

[49] Hsieh HL Kao CW Cheng SM Chang YC. A web-based integrated management program for improving medication adherence and quality of life, and reducing readmission in patients with atrial fibrillation: randomized controlled trial. J Med Internet Res. (2021) 23:e30107. doi: 10.2196/30107 34550084 PMC8495568

[50] Biesanz JC Falk CF Savalei V. Assessing mediational models: testing and interval estimation for indirect effects. Multivariate Behav Res. (2010) 45:661–701. doi: 10.1080/00273171.2010.498292 26735714

[51] Lopez-Jimenez F Almahmeed W Bays H Cuevas A Di Angelantonio E le Roux CW. Obesity and cardiovascular disease: mechanistic insights and management strategies. A joint position paper by the World Heart Federation and World Obesity Federation. Eur J Prev Cardiol. (2022) 29:2218–37. doi: 10.1093/eurjpc/zwac187 36007112

[52] Ma M Zhi H Yang S Yu EY Wang L. Body mass index and the risk of atrial fibrillation: A mendelian randomization study. Nutrients. (2022) 14(9). doi: 10.3390/nu14091878 PMC9101688 35565843

[53] Shu H Cheng J Li N Zhang Z Nie J Peng Y. Obesity and atrial fibrillation: a narrative review from arrhythmogenic mechanisms to clinical significance. Cardiovasc Diabetology. (2023) 22:192. doi: 10.1186/s12933-023-01913-5 PMC10387211 37516824

[54] Wanahita N Messerli FH Bangalore S Gami AS Somers VK Steinberg JS. Atrial fibrillation and obesity–results of a meta-analysis. Am Heart J. (2008) 155:310–5. doi: 10.1016/j.ahj.2007.10.004 18215602

[55] Blasco BV García-Jiménez J Bodoano I Gutiérrez-Rojas L. Obesity and depression: its prevalence and influence as a prognostic factor: A systematic review. Psychiatry Investig. (2020) 17:715–24. doi: 10.30773/pi.2020.0099 PMC7449839 32777922

[56] Fulton S Décarie-Spain L Fioramonti X Guiard B Nakajima S. The menace of obesity to depression and anxiety prevalence. Trends Endocrinol Metab. (2022) 33:18–35. doi: 10.1016/j.tem.2021.10.005 34750064

[57] Luppino FS de Wit LM Bouvy PF Stijnen T Cuijpers P Penninx BWJHc. Overweight, obesity, and depression: A systematic review and meta-analysis of longitudinal studies. Arch Gen Psychiatry. (2010) 67:220–9. doi: 10.1001/archgenpsychiatry.2010.2 20194822

[58] Gallucci M. jAMM: jamovi advanced mediation models. Available online at: https://jamovi-amm.github.io/ (Accessed January 17, 2023).

[59] The jamovi project (2022). Available online at: https://www.jamovi.org.

[60] Trivedi RB Blumenthal JA O'Connor C Adams K Hinderliter A Dupree C. Coping styles in heart failure patients with depressive symptoms. J Psychosom Res. (2009) 67:339–46. doi: 10.1016/j.jpsychores.2009.05.014 PMC2751656 19773027

[61] Zhao L Han G Zhao Y Jin Y Ge T Yang W. Gender differences in depression: evidence from genetics. Front Genet. (2020) 11:562316. doi: 10.3389/fgene.2020.562316 33193645 PMC7593575

[62] Hendriks JM Vrijhoef HJ Crijns HJ Brunner-La Rocca HP. The effect of a nurse-led integrated chronic care approach on quality of life in patients with atrial fibrillation. Europace. (2014) 16:491–9. doi: 10.1093/europace/eut286 24058179

[63] Hibbard JH Greene J. What the evidence shows about patient activation: better health outcomes and care experiences; fewer data on costs. Health Aff (Millwood). (2013) 32:207–14. doi: 10.1377/hlthaff.2012.1061 23381511

[64] Clarkesmith DE Pattison HM Khaing PH Lane DA. Educational and behavioural interventions for anticoagulant therapy in patients with atrial fibrillation. Cochrane Database Systematic Rev. (2017) 4. doi: 10.1002/14651858.CD008600.pub3 PMC6478129 28378924

[65] Sherwood A Blumenthal JA Koch GG Hoffman BM Watkins LL Smith PJ. Effects of coping skills training on quality of life, disease biomarkers, and clinical outcomes in patients with heart failure: A randomized clinical trial. Circ Heart Fail. (2017) 10(1). doi: 10.1161/circheartfailure.116.003410 PMC5233412 28062537

[66] Graven LJ Grant JS Vance DE Pryor ER Grubbs L Karioth S. Coping styles associated with heart failure outcomes: A systematic review. J Nurs Educ Practice. (2014) 4:227.

[67] Dornelas EA. Psychotherapy with cardiac patients: Behavioral cardiology in practice. Am psychol Assoc. (2008). doi: 10.1037/11809-000

[68] Soltani Shal R Aghamohammadian-Sharbaf H Abdekhodaie M-S Tayebi M. Effectiveness of Cardiovascular disease Specific Psychotherapy [CSP] on the stress, anxiety and depression of heart disease patients. Int J Behav Sci. (2016) 10:40–4.

[69] Sommaruga M. Cognitive and Behavioral Psychotherapy in Coronary Artery Disease. In: Roncella A Pristipino C, editors. Psychotherapy for Ischemic Heart Disease: An Evidence-based Clinical Approach. Cham, Switzerland: Springer International Publishing (2016). p. 159–72.

[70] Whalley B Thompson DR Taylor RS. Psychological interventions for coronary heart disease: cochrane systematic review and meta-analysis. Int J Behav Med. (2014) 21:109–21. doi: 10.1007/s12529-012-9282-x 23179678

[71] Fuenzalida C Hernandez G Ferro I Siches C Ambros A Coll-Vinent B. Long-term benefits of education by emergency care nurses at discharge of patients with atrial fibrillation. Int Emergency nursing. (2017) 35:7–12. doi: 10.1016/j.ienj.2017.03.006 28599914

[72] Bowyer JL Tully PJ Ganesan AN Chahadi FK Singleton CB McGavigan AD. A randomised controlled trial on the effect of nurse-led educational intervention at the time of catheter ablation for atrial fibrillation on quality of life, symptom severity and rehospitalisation. Heart Lung Circulation. (2017) 26:73–81. doi: 10.1016/j.hlc.2016.04.024 27423977

[73] Gagné M Legault C Boulet L-P Charbonneau L Lemyre M Giguere AMC. Impact of adding a video to patient education on quality of life among adults with atrial fibrillation: a randomized controlled trial. Patient Educ Counseling. (2019) 102:1490–8. doi: 10.1016/j.pec.2019.03.015 30956021

[74] van Munster KN van Mil J Safer R Nieuwkerk PT Ponsioen CY. Improving disease knowledge of primary sclerosing cholangitis patients and their relatives with a 3-dimensional education video. Patient Educ Couns. (2020) 103:960–4. doi: 10.1016/j.pec.2020.01.005 32005555

[75] Li N Yao X Ji H. Relationships among disease knowledge, social support, anxiety and self-efficacy in patients after total knee arthroplasty: A chain mediating effect. Nurs Open. (2023) 10:4728–36. doi: 10.1002/nop2.1723 PMC10277396 36879531

[76] Zemni I Gara A Nasraoui H Kacem M Maatouk A Trimeche O. The effectiveness of a health education intervention to reduce anxiety in quarantined COVID-19 patients: a randomized controlled trial. BMC Public Health. (2023) 23:1188. doi: 10.1186/s12889-023-16104-w 37340300 PMC10280925

[77] Tam W Woo B Lim TW. Questionnaires designed to assess knowledge of atrial fibrillation: A systematic review. J Cardiovasc Nurs. (2019) 34:E14–e21. doi: 10.1097/jcn.0000000000000576 30932924

[78] Kramer Freeman L Richards K Conti JB Sears SF. Patients with implantable cardioverter defibrillators on social media report more shock anxiety than clinic patients: results from an online survey. JMIR Cardio. (2017) 1:e6. doi: 10.2196/cardio.8152 31758785 PMC6857955

[79] (NICE). NIfHaCE. Atrial fibrillation: diagnosis and management (2023). Available online at: https://www.nice.org.uk/guidance/ng196/chapter/Recommendations (Accessed 27 June 2023).

[80] January CT Wann LS Calkins H Chen LY Cigarroa JE Cleveland JC. 2019 AHA/ACC/HRS focused update of the 2014 AHA/ACC/HRS guideline for the management of patients with atrial fibrillation: A report of the american college of cardiology/american heart association task force on clinical practice guidelines and the heart rhythm society in collaboration with the society of thoracic surgeons. Circulation. (2019) 140:e125–51. doi: 10.1161/CIR.0000000000000665 30686041

